# Dental health problems and treatment-seeking behavior among special need school students in Amhara region, Ethiopia

**DOI:** 10.1186/s12903-021-01856-x

**Published:** 2021-10-02

**Authors:** Amare Teshome Tefera, Biruk Girma, Aynishet Adane, Abebe Muche, Tadesse Awoke Ayele, Kefyalew Ayalew Getahun, Zelallem Aniley, Semira Ali, Simegnew Handebo

**Affiliations:** 1 Department of Dentistry, School of Medicine, College of Medicine and Health Sciences, University of Gondar, Gondar, Ethiopia; 2 Department of Internal Medicine, School of Medicine, College of Medicine and Health Sciences, University of Gondar, Gondar, Ethiopia; 3 Department of Anatomy, School of Medicine, College of Medicine and Health Sciences, University of Gondar, Gondar, Ethiopia; 4 Department of Epidemiology and Biostatistics, Institute of public health, College of Medicine and Health Sciences, University of Gondar, Gondar, Ethiopia; 5 Department of Pharmacology, School of Pharmacy, College of Medicine and Health Sciences, University of Gondar, Gondar, Ethiopia; 6 Department of Special Needs and Inclusive Education, College of Education, University of Gondar, Gondar, Ethiopia; 7 Department of Health Education and Behavioral Sciences, Institute of Public Health, College of Medicine and Health Sciences, University of Gondar, P.O.Box-196, Gondar, Ethiopia

**Keywords:** Dental health, Dental treatment-seeking, Special needs, Ethiopia

## Abstract

**Background:**

Oral diseases are a public health concern with a significant impact on the quality of life of individuals. Children with special needs face significant challenges in carrying out oral hygiene due to their disability, and they are more prone to poor oral health and illnesses. This study assessed dental health problems and treatment-seeking behaviors of special needs school students in Amhara region, Ethiopia.

**Methods:**

An institution-based cross-sectional study was conducted from November 2020 to April 2021, in eight special needs schools located in the Amhara Regional State, Ethiopia. A total of 443 randomly selected special needs students were included. Data were collected using a structured interview-administered questionnaire. Bivariable and multivariable logistic regression models were fitted to identify factors associated with oral health problems and treatment-seeking behavior. A *p*-value of less than 0.05 was used to declare statistical significance.

**Results:**

The prevalence of self-reported dental health problems and treatment-seeking behaviors among special needs school students was 46.1% (95% CI: 41.4%, 50.7%) and 60.3% (95% CI: 53.4%, 66.8%), respectively. Place of residence, grade level, religious affiliation, years lived with disability, and knowledge of dental health-related risk behaviors were associated with dental health problems. Whereas, place of residence, being hearing impaired, and having prior information about dental health problems were associated with dental treatment-seeking behavior.

**Conclusions:**

A significant number of special needs students reported dental problems and about 40% of them did not seek dental treatment. Oral hygiene practice and access to dental care services are important in the prevention of dental problems. Hence, oral hygiene promotion programs focusing on oral hygiene practice and dental treatment services are needed in special needs schools. It is also strongly suggested to incorporate oral health related information in health-related academic lessons to enhance optimum oral health among special needs students.

## Background

Globally, it is estimated that over 1 billion people live with some form of disability, accounting for approximately 15% of the world’s population. Among this, 93 million children and 720 million adults live with severe disabilities [1]. In Ethiopia, the proportion of people with disabilities is estimated to be around 17.6%, implying that more than 14.4 million Ethiopians live with a disability, including 2.5 million children [2].

Oral diseases disproportionally affect the poor and socially disadvantaged members of society [3]. Disabled individuals appear to have poorer oral health than their non-disabled counterparts [4]. A systematic review on the oral health status reported that children and adolescents with intellectual disabilities have poorer oral health (higher level of dental plaque, worse gingival status, and fewer decayed and filled permanent teeth) compared to their counterparts [5]. According to the Global Burden of Disease, untreated dental caries in permanent teeth is the most prevalent oral health problem [6], and with over 530 million children suffering from primary tooth caries [3]. Children with disabilities and other special needs have more oral health problems than the general population due to impaired cognitive abilities, behavioral problems, impaired mobility, and neuromuscular problems. They require extra help and rely on others to achieve and maintain good oral health [7].

Article 25 of the UN Convention on the Rights of Persons with Disabilities (CRPD) reinforces the right of persons with disabilities to attain the highest standard of healthcare, without discrimination [8]. However, the numbers of children with disabilities have steadily increased with demographic trends; most health systems cannot even address the current needs of children with disabilities. The unmet needs of children with disabilities have increased considerably, as health services have not expanded to meet the growing need [9]. Most low- and middle-income countries are unable to provide services to prevent and treat oral health conditions [3].

Oral diseases pose a major health burden for many countries and affect people throughout their lifetime, causing pain, discomfort, disfigurement, and even death [3]. People with a disability may have worse oral health than those without disabilities; this might not only cause physical problems, but it can also have a far-reaching impact since poor oral health can hurt self-esteem, quality of life, and general health [10]. Moreover, oral diseases and conditions have serious health and economic burden, particularly among school-age children and adolescents [11].

Healthcare-seeking behavior is, any action taken by individuals who believe that they have a health problem or believe that they are ill [12]. It comprises activities carried out to maintain good health, to prevent ill health, as well as any departure from a good state of health [13]. Oral healthcare-seeking seems to be inextricably linked to the demand for oral healthcare services[14]. Thus, the demand for oral healthcare services is often associated with an individual’s choice about which service to access and when and where to access healthcare services [15]. Inadequate health-seeking behavior has been associated with poorer oral health outcomes, higher mortality & morbidity, and lower oral health statistics [16]. Knowing the oral health problems and challenges of dental care utilization among disabled individuals has the utmost effect to design interventions. To the best of our knowledge, there is no documented evidence of dental health problems and treatment-seeking behavior among schoolchildren in Ethiopia. Hence, the purpose of this study was to assess the dental health problems and treatment-seeking behavior of special needs school students in in Amhara regional state, Ethiopia.

## Methods

### Study area and period

A cross-sectional study was conducted from November 2020 to April 2021 in eight special needs schools in the Amhara Regional State, Ethiopia: Gondar, Dessie, Debre Markos, and Bahir Dar. In the study area, 696 disabled students are attending special needs schools (Gondar = 170, Dessie = 179, Bahir Dar = 237, and Debre Markos = 110). The disability type distribution revealed that 341 were hearing impaired, 129 were visually impaired and 226 were mentally handicapped.

### Population

The study participants were disabled students attending special needs education special in Amhara Region, Ethiopia. Students who were absent throughout the data collection period, unable to provide complete data, and severely ill were excluded from the study.

### Sample size and sampling procedure

The sample size was calculated using a single population proportion formula; considering 50% (since no previous study found in Ethiopia) proportion of students who have a dental health problem, d (the permissible Margin of error 5%), Zα/2 (the value of the standard normal curve score corresponding to the given confidence interval = 1.96) corresponding to 95% confidence level, and 15% non-response rate. The final sample size was estimated to be 443 participants. The list of special need students was obtained from each school class roaster. A simple random sampling technique using a computer random number generator was employed to recruit the study participants.

### Data collection procedure

Data were collected using a pretested structured interview-administered questionnaire adapted from the WHO oral health survey tool and other literature [10, 15, 17, 18]. In the very beginning, the tool was prepared in English and then translated into the local Amharic language. To check the consistency of the questionnaire, the Amharic version was translated back to English. Pretest was done on 5% of the total sample size and modifications were made accordingly. The final instrument was composed of socio-demographic characteristics, oral health practice, medical condition, disability, oral habits, and dental care-seeking behaviors.

The data collection and supervision were done by qualified dental professionals and special needs experts. Data collectors and supervisors received a five-day training on the purpose of the study, data collection techniques, and ethical considerations during data gathering. Daily, each returned questionnaire was checked for completeness and consistency.

### Data processing and analysis

The data were entered into EpiData version 4.6 and exported into STATA version 14 statistical software for analysis. Descriptive analyses like medians, means, proportions, standard deviations, and frequencies were computed. A bivariable and multivariable logistic regression model was fitted to identify the factors associated with dental health problems and treatment-seeking behavior among special needs school students in Amhara region. Those variables with a p-value of less than 0.25 in the bivariable model were fitted in the multivariable model. Variables with a p-value less than 0.05 at a 95% confidence interval were considered statistically significant.

## Results

### Socio-demographic characteristics

A total of 443 special needs students participated in the study with a response rate of 100%. The mean age of participants was 15.84 (SD ± 0.18 years) with the age range of 7 to 30 years. More than half of the participants were males (53.5%) and attending primary education (53.3%). The higher proportion of the participants (69.8%) were affiliated with Orthodox religion. The monthly income of the majority of the participants (70.5%) was below 1000 Ethiopian Birr (Table 1**)**.

**Table 1:** Socio-demographic characteristics of special need students in Amhara region, Ethiopia, 2021

Variables	Category	Male n (%)	Female n (%)	Total n (%)
Age	Below 18 years	151 (63.71)	165 (80.10)	316 (71.33)
	18 and above years	86 (36.29)	41 (19.90)	127 (28.67)
Location	Gondar	49 (20.68)	43 (20.87)	92 (20.77)
	Bahir Dar	79 (33.33)	65 (31.55)	144 (32.51)
	Debre Markos	75 (31.65)	58 (28.16)	133 (30.02)
	Dessie	34 (14.35)	40 (19.42)	74 (16.70)
Grade	1–4 grade	120 (50.63)	116 (56.31)	236 (53.27)
	5–8 grade	77 (32.49)	72 (34.95)	149 (33.63)
	9–12 grade	40 (16.88)	18 (8.74)	58 (13.09)
Religious	Orthodox	173 (73.00)	136 (66.02)	309 (69.75)
	Catholic	33 (13.92)	30 (14.56)	63 (14.22)
	Muslim	27 (11.39)	35 (16.99)	62 (14.00)
	Protestant	4 (1.69)	5 (2.43)	9 (2.03)
Mother educational status	No education	154 (66.96)	103 (53.65)	257 (60.90)
	Able to read and write	48 (20.87)	65 (33.85)	113 (26.78)
	Formal education	28 (12.17)	24 (12.50)	52 (12.32)
Father educational status	No education	114 (50.22)	83 (43.46)	197 (47.13)
	Able to read and write	73 (32.16)	69 (36.13)	142 (33.97)
	Formal education	40 (17.62)	39 (20.42)	79 (18.90)
Family income	Less than 1000 ETB	152 (70.70)	123 (70.29)	275 (70.51)
	1000–2500 ETB	39 (18.14)	29 (16.57)	68 (17.44)
	Greater than 2500 ETB	24 (11.16)	23 (13.14)	47 (12.05)

*ETB* Ethiopian Birr

### Oral hygiene practice

More than three-quarters (76.1%) of study participants had a tooth brushing habit, and nearly half (53.1%) of them used toothbrushes when brushing their teeth. About 16.7% of the participants brushed their teeth twice or more times a day. Furthermore, about 17.4% of the participant received support from parents or legal guardians while brushing their teeth.

### Type of disabilities

One-third of the participants (33.6%) had hearing impairment, and 29.4% had visual impairment. The median (inter-quartile range) of years lived with a disability was 14 (12–16 years).

### Self-reported oral health problems and dental care-seeking behavior

Of the total study participant, 204 (46.1%, 95% CI: 41.4%, 50.7%) reported oral health problems. From these, 123 (60.3%, 95% CI: 53.4%, 66.8%) sought dental care within the median time of 5 days, interquartile range of (5–7days). The majority (65.0%) of the participant sought dental care at the serious stage of the disease. More than half (53.7%) of the participants followed the dental care treatment courses until recovery. Fear (29.7%) and cost of the treatments (29.7%) were the two main barriers not to sought dental care. On the other hand, nearly half (48.1%) of the participants did not know dental health-related risky behavior (Table 2).

**Table 2:** Oral health problems and dental care-seeking behavior of special need students in Amhara region, Ethiopia, 2021

Variables	Category	Male n (%)	Female n (%)	Total n (%)
Heard about dental diseases	Yes	176 (74.26)	139 (67.48)	315 (71.11)
	No	61 (25.74)	67 (32.52)	128 (28.89)
Source of information about dental problem	Family members	117 (67.24)	104 (74.82)	221 (70.61)
	Media	37 (21.26)	14 (10.07)	51 (16.29)
	Health professionals	14 (8.05)	17 (12.23)	31 (9.90)
	Others	6 (3.45)	4 (2.88)	10 (3.19)
Risk behaviors for dental health problem	Don’t know	111 (46.84)	102 (49.51)	213 (48.08)
	Poor oral hygiene	83 (35.02)	67 (32.52)	150 (33.86)
	Poor oral hygiene and taking sugary food	29 (12.24)	16 (7.77)	45 (10.16)
	Taking sugary food	14 (5.91)	21 (10.19)	35 (7.90)
Self-reported dental health	Yes	108 (45.57)	96 (46.60)	204 (46.05)
	No	129 (54.43)	110 (53.40)	239 (53.95)
Dental care seeking	Yes	63 (58.33)	60 (62.50)	123 (60.29)
	No	45 (41.67)	36 (37.50)	81 (39.71)
Stage of the disease during dental care	Early stage	8 (12.70)	6 (10.00)	14 (11.38)
	Serious stage	42 (66.67)	38 (63.33)	80 (65.04)
	I don’t know	13 (20.63)	16 (26.67)	29 (23.58)
Course of the treatment followed	To recover	33 (52.38)	33 (55.00)	66 (53.66)
	To relieve from the symptoms	16 (25.40)	18 (30.00)	34 (27.64)
	Do not completed	14 (22.22)	9 (15.00)	23 (18.70)
Barriers not to seek dental care	Fear	14 (33.33)	8 (25.00)	22 (29.73)
	Cost of the treatment	14 (33.33)	8 (25.00)	22 (29.73)
	Lack of knowledge	9 (21.43)	11 (34.38)	20 (27.03)
	Others*	5 (11.90)	5 (15.63)	10 (13.51)

### Perception towards dental care services

**Fig. 1:**
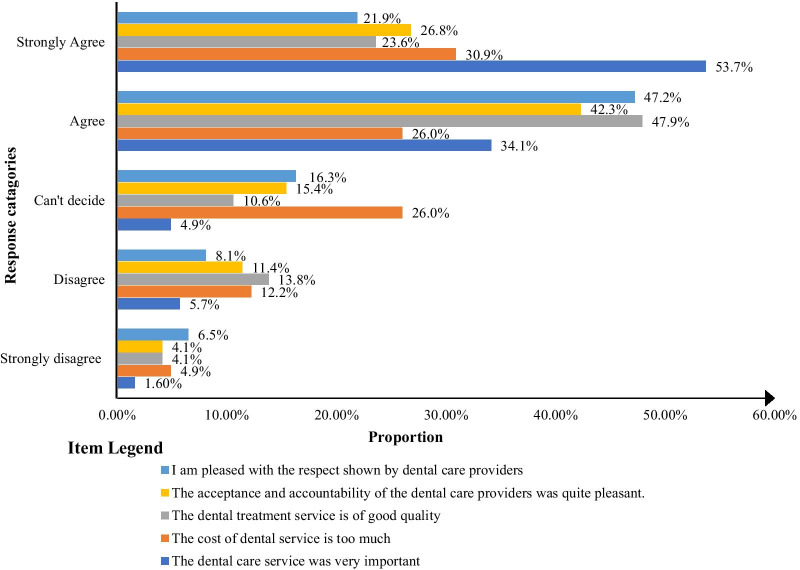
Perception towards dental care services among special need students in Amhara region, Ethiopia, 2021

The study participants’ perception of dental care service was evaluated using five items with a five-point Likert scale. About 88% and 72% of the participants thought dental care was important and the quality of the care was good, respectively. Besides that, the cost of dental care was too high for more than half of the participants (56.9%). Majority (69.1%) of the participants were pleased by the respect and behavior of dental professionals (Fig. 1).

### Treatment need

**Fig. 2:**
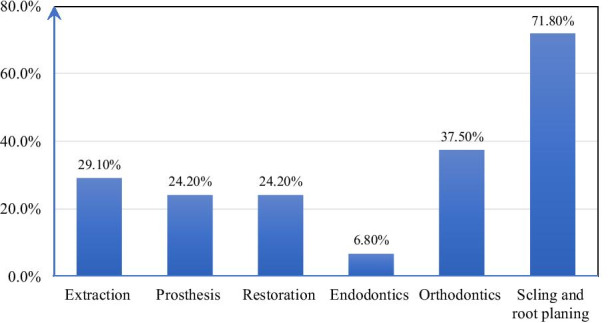
Dental treatment need of special need students in Amhara region, Ethiopia, 2021

Concerning treatment needs, the majorities (71.8%) of the participants require scaling and root planning and more than one-third (37.8%) of them require Orthodontics treatments (Fig. 2).

### Factors associated with dental health problems

As presented in Table 3, place of residence, grade level, maternal educational status, paternal educational status, religion, carbohydrate intake, frequency of tooth brushing, types of disability, years lived with disability, knowledge on risk behaviors for oral health, and comorbidity was variables with a *p*-value of less than 0.25 in a bivariable logistic regression model. These variables were entered into the final multivariable logistic regression model. Of them, place of residence, grade level, religious affiliation, years lived with disability, and knowledge of dental health-related risk behaviors were statistically significant factors associated with dental health problems.

Students who lived in Bahir Dar (AOR = 0.32, 95% CI: (0.15, 0.70)) and Debre Markos (AOR = 0.45, 95% CI: (0.22, 0.93)) cities were less likely to have dental health problems as compared to those who lived in Gondar. Students attending grades 5–8 (AOR = 2.04 95% CI: (1.14, 3.65)) and grades 9–12 (AOR = 4.25 95% CI: (1.91, 9.47)) were more likely had dental health problems than students attending grade 1–4 did. Students affiliated with the Islamic religion were 2.38 times more likely to report dental health problems compared to those affiliated with Orthodox Christians (AOR = 2.38, 95% CI: (1.07, 5.32)). Students with mental impairment were 2.42 times more likely to report dental health problems compared to visually impaired students (AOR = 2.42, 95 %nCI: (1.14, 5.11)). Students who knew at least one dental health-related risky behavior were 2.31 more likely to report dental health problems compared to their counterparts (AOR = 2.31, 95% CI: (1.40, 3.80)). The risks of having oral health problems drop by 0.92 times for every year spent living with a disability (AOR = 0.92, 95% CI: (0.87, 0.98) (Table 3).

**Table 3:** Factors associated with dental health problems among special need students in Amhara region, Ethiopia, 2021 (n = 443)

Independent variables		Dental health problem		COR (95% CI)	AOR (95% CI)
		No	Yes		
Residence	Gondar	45 (48.9)	47 (51.1)	1	1
	Bahir Dar	95 (66.0)	49 (34.0)	0.49 (0.29, 0.84)	0.32 (0.15, 0.70)**
	Debre Markos	76 (57.1)	57 (42.9)	0.72 (0.42, 1.22)	0.45 (0.22, 0.93)*
	Dessie	23 (31.1)	51 (68.9)	2.12 (1.12, 4.03)	0.84 (0.35, 2.04)
Grade	1–4 grade	139 (58.9)	97 (41.1)	1	1
	5–8 grade	73 (49.0)	76 (51.0)	1.49 (0.99, 2.25)	2.04 (1.14, 3.65)*
	9–12 grade	27 (46.5)	31 (53.5)	1.64 (0.92, 2.93)	4.25 (1.91, 9.47)**
Religion	Orthodox	190 (61.5)	119 (38.5)	1	1
	Catholic	27 (42.9)	36 (57.1)	2.13 (1.23, 3.69)	1.34 (0.64, 2.82)
	Muslim	17 (27.4)	45 (72.6)	4.23 (2.31, 7.73)	2.38 (1.07, 5.32)*
	Protestant	5 (55.6)	4 (44.4)	1.28 (0.34, 4.85)	1.30 (0.26, 6.47)
Mother educational status	No education	152 (59.1)	105 (40.9)	1	1
	Able to read and write	49 (43.4)	64 (56.6)	1.89 (1.21, 2.96)	1.61 (0.88, 2.94)
	Formal education	29 (55.8)	23 (44.2)	1.15 (0.63 2.09)	0.98 (0.40, 2.42)
Father educational status	No education	114 (57.9)	83 (42.1)	1	1
	Able to read and write	72 (50.7)	70 (49.3)	1.34 (0.87, 2.06)	1.20 (0.70, 2.07)
	Formal education	43 (54.4)	36 (45.6)	1.15 (0.68,1.94)	1.01 (0.45, 2.25)
Taking sugared foods	Yes	222 (54.9)	182 (45.1)	1	1
	No	17 (43.6)	22 (56.4)	1.58 (0.81, 3.06)	1.36 (0.60, 3.11)
Frequency of tooth brushing	Never	58 (54.7)	48 (45.3)	1	1
	Sometimes	105 (56.8)	80 (43.2)	0.92 (0.57, 1.49)	0.68 (0.37, 1.26)
	Once a day	53 (45.3)	64 (54.7)	1.46 (0.86, 2.47)	1.07 (0.56, 2.07)
	Twice a day	23 (65.7)	12 (34.3)	0.63 (0.28, 1.40)	0.50 (0.19, 1.28)
Disability types	Visually impaired	72 (55.4)	58 (44.6)	1	1
	Hearing impaired	69 (46.3)	80 (53.7)	1.44 (0.90, 2.31)	1.65 (0.92, 2.97)
	Mental impaired	84 (61.3)	53 (38.7)	0.78 (0.48, 1.28)	2.42 (1.14, 5.11)*
	Others	14 (51.8)	13 (48.2)	1.15 (0.50, 2.64)	0.99 (0.35, 2.78)
Comorbidity	Yes	20 (35.1)	37 (64.9)	1	1
	No	219 (56.7)	167 (43.3)	0.41 (0.23, 0.74)	0.66 (0.32, 1.36)
Knowledge of risk behaviors for oral health	No	139 (65.3)	74 (34.7)	1	1
	Know at least one	100 (43.5)	130 (56.5)	2.44 (1.66, 3.59)	2.31 (1.40, 3.80)**
Years lived with disability^&^ Mean (SD)		14.6 (± 4.2)	13.8 (± 3.8)	0.95 (0.90, 0.99)	0.92 (0.87, 0.98)**

^&^Continuous variable *Significant at *p* value < 0.05, **Significant at *p*-value < 0.01

### Factors associated with dental treatment-seeking behavior

The multivariable analysis showed that living in Dessie town, being hearing impaired, and having prior information about dental health problems were statistically significant factors associated with dental treatment-seeking behavior. Students who lived in Dessie town were 4.48 times more likely to seek dental treatments than students who lived in Gondar city (AOR = 4.48, 95% CI: (1.10, 18.25)). Hearing-impaired students were 3.70 times more likely to seek dental treatments compared to visually impaired students (AOR = 3.70, 95% CI: (1.46, 9.43)). Moreover, students who did not have prior information about dental health problems were 86% less likely to seek dental treatments than their counterparts (AOR = 0.14, 95% CI: (0.04, 0.44)) (Table 4).

**Table 4:** Factors associated with dental treatment seeking behavior among special need students in Amhara region, Ethiopia, 2021 (n = 204)

Independent variables		Treatment seeking		COR (95% CI)	AOR (95% CI)
		No	Yes		
Age of the participant^&^ (mean ± SD)		16.5 (± 3.8)	15.6 (± 3.5)	0.93 (0.86, 1.01)	0.92 (0.81, 1.04)
Residence	Gondar	23 (48.9)	24 (51.1)	1	1
	Bahir Dar	25 (51.0)	24 (49.0)	0.92 (0.41, 2.05)	0.52 (0.13, 2.07)
	Debre Markos	26 (45.6)	31 (54.4)	1.14 (0.53, 2.48)	0.76 (0.22, 2.68)
	Dessie	7 (13.7)	44 (86.3)	6.02 (2.26, 16.07)	4.48 (1.10, 18.25)*
Grade	1–4 grade	43 (44.3)	54 (55.7)	1	1
	5–8 grade	25 (32.9)	51 (67.1)	1.62 (0.87, 3.03)	2.17 (0.80, 5.87)
	9–12 grade	13 (41.9)	18 (58.1)	1.10 (0.49, 2.50)	3.38 (0.82, 13.92)
Religion	Orthodox	55 (46.2)	64 (53.8)	1	1
	Catholic	13 (36.1)	23 (63.9)	1.52 (0.70, 3.28)	0.83 (0.25, 2.76)
	Muslim	12 (26.7)	33 (73.3)	2.36 (1.11, 5.02)	1.03 (0.34, 3.11)
	Protestant	1 (25.0)	3 (75.0)	2.58 (0.26, 25.5)	1.65 (0.11, 25.16)
Mother educational status	No education	49 (46.7)	56 (53.3)	1	1
	Able to read and write	21 (32.8)	43 (67.2)	1.79 (0.94, 3.42)	1.29 (0.48, 3.45)
	Formal education	8 (34.8)	15 (65.2)	1.64 (0.64, 4.20)	1.26 (0.30, 5.26)
Father educational status	No education	35 (42.2)	48 (57.8)	1	1
	Able to read and write	23 (32.9)	47 (67.1)	1.49 (0.77, 2.89)	2.15 (0.82, 5.62)
	Formal education	16 (44.4)	20 (55.6)	0.91 (0.41, 2.01)	1.18 (0.33, 4.19)
Disability types	Visually impaired	30 (51.7)	28 (48.3)	1	1
	Hearing impaired	22 (27.5)	58 (72.5)	2.82 (1.39, 5.75)	3.70 (1.46, 9.43) **
	Mental impaired	25 (47.2)	28 (52.8)	1.20 (0.57, 2.53)	2.64 (0.83, 8.46)
	Others	4 (30.8)	9 (69.2)	2.41 (0.67, 8.72)	5.85 (0.87, 39.14)
Comorbidity	Yes	9 (24.3)	28 (75.7)	1	1
	No	72 (43.1)	95 (56.9)	0.42 (0.19, 0.95)	1.64 (0.08, 32.89)
Medication intake	Yes	9 (26.5)	25 (73.5)		
	No	72 (42.4)	98 (57.6)	0.49 (0.22, 1.11)	0.40 (0.02, 8.16)
Heard about dental problem	Yes	59 (33.7)	116 (66.3)	1	1
	No	22 (75.9)	7 (24.1)	0.16 (0.07, 0.40)	0.14 (0.04, 0.44)**

^&^Continuous variable *Significant at *p* value < 0.05 **Significant at *p*-value < 0.01

## Discussion

This study was aimed to assess the dental health problems and treatment-seeking behavior of special needs school students in Amhara Regional State, Ethiopia. Individuals with disabilities appear to have poorer oral health than their non-disabled counterparts [4]. In support of this, the present study revealed that 46.1% of special needs students reported dental health problems. A similar finding was reported in Canada among people experiencing social and health inequalities where 46.3% of the participants had poor oral health [19]. On the contrary, Choi and Yang reported lower caries prevalence among children with disabilities compared with those without disabilities [20]. In general, increased urbanization and changes in living conditions could be a contributing factor for the high prevalence of oral health problems [3]. Moreover, children with special needs have more oral health problems due to existing condition-related complications that can be barriers to adequate oral hygiene practice [7]. Physical limitations that make tooth brushing difficult, reduced saliva flow, taking medications and precarious diets are reported as factors that contribute to poor oral health in people with disabilities [21]. Due to this, students with disabilities depend on others to achieve and maintain good oral health [21]. This reflects that ongoing coaching and reinforcement from caregivers is critical in improving special needs students’ oral care practices and reducing the occurrence of oral health problems [22]. However, the present study found that about 82% of the participant did not get adequate support from their family members to do so.

In this study living in Bahir Dar and Debre Markos cities, and increased years lived with disability were associated with lower dental health problems. On the other hand, attending grades 5–8 and grades 9–12, and being affiliated Islam religion was associated with increased dental health problems. These findings may be attributed to differences in geographic location, socio-economic, and other confounding factors. A previous study reported that children with hearing impairment had lower caries experience than children with mental retardation and visual impairment [4]. Besides in this study knowledge of dental risky behaviors was negatively associated with a dental health problem. In such cases, the knowledge by itself may not enable us to predict the behaviors. This means individuals who know dental health-related risky behaviors may not adhere to them appropriately. This may be due to knowledge is neither sufficient nor necessary to trigger a behavioral change [23].

Meeting the oral health treatment requirements for children with intellectual, emotional, or physical disabilities can be a difficult task for their caregivers and health professionals [24]. Children with special needs, oral health needs are competing with already burdensome chronic health conditions [25]. Oral health treatment-seeking and decision about what type of treatment to receive could be said to depend on the recommendations of family and friends and the cost of services [16]. In the present study, the prevalence of dental treatment-seeking was 60.3%. Similarly, a study on oral health care services utilization among children in Lagos, Nigeria, found that children with disabilities did not adequately use dental facilities [25]. Children’s treatment-seeking behavior was attributed to family’s low commitment to their dental care. A national survey of children with special health care needs in the USA from 2005 to 2011 found that children with special health care needs are experiencing greater unmet dental needs and are receiving less help coordinating care services [26].

The analysis of factors associated with dental treatment-seeking behavior revealed that children living in Dessie town, hearing impaired, and who had prior information about dental health problems had higher dental treatment-seeking behavior. A cross-sectional study on the oral health-seeking behavior of different population groups in Nigeria found that geographic location and socio-economic status group have a negative regression coefficient to the demand for treatment in the dental clinics. Any movement towards the urban area will increase the demand for dental caries treatment in a dental facility [16]. This is may be due to differences in access to dental health services and information, and geographical location of the participants and services. A systematic review reported that people with disabilities encounter physical, structural, geographical, professional, or behavioral barriers that hinder access to dental services. Specifically, the most frequently reported barriers to use dental care among disabled peoples were the cost of treatment, lack of preparation for dental care of the disabled persons, the inadequacy of dental facilities for the disabled, and lack of adaptation of the access routes to the health care facilities and dental offices [27]. This indicates the need for dental centers accessible to special needs individuals, and trained dental professionals [28].

Despite some limitations, the present study tried to assess an important public health issue in marginalized populations and provide a way for successive researchers. This is the first study on the dental problem and treatment-seeking practice of special needs school students in Ethiopia. The first limitation, the findings were based on self-reported data and may be subject to social desirability bias. Secondly, due to the impairment, some of the study participants did not express their dental problems confidentially. This means we have used language translators for hearing-impaired participants. Finally, due to the cross-sectional nature of the study, the causal relationship between the dependent and independent variables cannot be declared.

## Conclusion

The present study found that a significant proportion of special needs school students had oral health problems. In addition, a significant number of them did not seek dental treatments. Dental health problems and dental treatment-seeking behavior of special need school students were associated with a variety of child and familial socio-demographic characteristics, types of disabilities, years lived with disabilities, knowledge of oral health-related risk behaviors, frequency of carbohydrate intake, and information about dental health problems. Therefore, schools and centers for special needs students should develop and implement oral hygiene programs focusing on screening, prevention, and treatment of oral health problems to reduce the impact of dental diseases. Policymakers, health professionals, and other concerned bodies should emphasize oral health care as a major component of the overall wellbeing of children with special needs. On the other hand, oral health promotion programs and tailored oral health education programs focusing on oral hygienic practices, risky behaviors, dental diseases preventive strategies, and dental treatment-seeking behaviors are recommended to achieve optimum oral health. Finally, it is also strongly suggested to incorporate oral health-related messages/information in health-related academic lessons.

## Data Availability

All datasets related to this article will be available upon a reasonable request from the corresponding author (Simegnew Handebo, E-mail: simegnewh@gmail.com).

## Abbreviations

AOR: Adjusted odds ratio
CI: Confidence interval
COVID: Coronavirus disease
CRPD: Convention on the rights of persons with disabilities
COR: Crude odds ratio
SD: Standard deviation
WHO: World Health Organization
